# The Settlement of Disputes

**Published:** 1915-10-09

**Authors:** 


### NATIONAL INSURANCE ACT DEVELOPMENTS.

### The Settlement of Disputes.

Special provision is made under the National Insurance Acts for the settlement of disputes between insured persons and their approved societies or Insurance Committees, and of disputes between approved societies and Insurance Committees. The procedure involves the submission of the matters in dispute in the first instance to arbitrators, but the right of appeal to the Insurance Commissioners is allowed in the event of either party being dissatisfied with the result of the arbitration. When such appeals are made to the Commissioners they appoint referees to hear the evidence, and the decision of such referees is final.

This procedure was designed to obviate the necessity of applications to Courts, and thus to minimise the cost of the settlement of disputes. The object has undoubtedly much to commend it, but there is one serious disadvantage in the system—namely, that as the arbitration proceedings and the hearing before the referees are generally, if not always, conducted in private, the decisions are not given the publicity which they would secure if they were delivered in Court. For this reason the Insurance Commissioners have thought it advisable to publish the decisions given by the referees in order that the principles enunciated may become known, and thus facilitate the settlement of disputes of a like nature. The first report of such decisions was published in March last. This has been quickly followed by a second report,* published last month, in which are recorded the decisions arrived at in twenty-four cases which were heard between October 1914 and July 1915.

The whole of the report is interesting and instructive to those whose duty it is to administer the National Insurance Acts, but most of the cases refer to disputes regarding the rights of insured persons to sickness benefit in certain special circumstances, and they are accordingly of little general interest. There are, however, two cases which appear to us to merit attention.

### Medical Benefit—Permission to make Own Arrangements.

The first of these cases arises out of a dispute between an insured person and the Insurance Committee for the area in which he resided, and involves the question of the right of an insured person to be given permission to make his own arrangements for the receipt of medical benefit.

The summary printed at the head of the decision of the referees is as follows: "An insured person applied for permission to make his own arrangements for medical benefit. The grounds of his application were that he desired to be attended by a medical practitioner not on the panel for the area in which he resided, who had attended him before medical benefit began (i.e., January 1913), and was actually treating him at that date. The applicant alleged that it would be detrimental to his health to be compelled to change to another doctor. The Insurance Committee did not dispute the *bona fides* of the applicant's request nor any of the facts on which he relied as supporting it, but rejected it on the ground that it was still open to the doctor in question to come on the panel, and that the nature of the applicant's illness was not such as to require special treatment. The referees held that the Insurance Committee had come to an unreasonable decision, and had misdirected themselves with regard to the reasons for their refusal, and that the applicant was entitled to such contribution from the Insurance Committee towards his medical attendance and treatment as he would have obtained had he been granted permission to make his own arrangements for medical attendance and treatment." This summary is as clear and concise as possible, but it is necessary to point out that in their decision the referees stated quite plainly that while the Insurance Committee was under no obligation to grant permission to any insured person to make his own arrangements, merely because he choose to ask for it, it was essential that the committee should exercise the discretion reposed in them in a reasonable manner, and after due consideration of all the circumstances of the case. In this connection it was pointed out that regard should be had primarily to the interests of the insured person making the application; but it was also necessary to take into account the interests of the medical service of insured persons generally within their area. For the latter reason the referees held that it was reasonable that any insured person desiring to make his own arrangements for medical benefit—that is to say, for securing the services of a doctor not on the panel—should show cause why his case should be treated in an exceptional way. Having, however, been furnished with the reasons for asking permission, the Insurance Committee must act in a reasonable manner.

### An Insurance Committee Criticised.

The referees proceeded to criticise the attitude of the particular Insurance Committee, by asking how it could be regarded as a sufficient reason for their refusal that it was still open to the doctor to come upon the panel if he thought fit to do so. They also doubted whether the opinion of the committee that the illness of the applicant was not such as to require special treatment was a relevant consideration.

"The confidence between patient and doctor, which is admittedly a most important element in successful treatment, ought not to be arbitrarily restricted to the case of the more serious illnesses, nor the rights of an insured person to vary with the state of his health."

It will be admitted that this is a most important case which, although it may not be taken as a precedent, is an indication that the Insurance Com-

* Reports of Decisions on Appeals and Applications under National Insurance Acts. Part II. Cd. 8040. 3½d.

mittees must treat with fairness and consideration applications made to them by those who are not content to obtain medical treatment and attendance from a panel doctor.

Payment to Insurance Committee for Sanatorium Inmate without Dependants.

The other case which we think should be noted refers to an application made by an Insurance Committee for the payment of sickness benefit in respect of an insured person, without dependants, admitted for treatment in a sanatorium. The approved society against whom the claim was made resisted payment on the ground that incapacity for work had not been proved. As a matter of fact the insured member had, up to the time of her admission to the sanatorium, been at work, and she resumed her employment immediately on her discharge. The referees held, however, that the right of an Insurance Committee to claim the sum which would have been payable on account of sickness benefit, if the insured person had not been an inmate in a sanatorium, was based upon an independent statutory title, and consequently it was not necessary that the precise formalities which would have had to be observed if the member had claimed should be observed when the claim was made by the committee. The Insurance Committee were, nevertheless, bound to show that the insured person has been rendered incapable of work by some specific disease or bodily or mental disablement of which notice has been given.

An insured person whose state of health is such that he has become, on the recommendation of an Insurance Committee, an inmate of a sanatorium, must necessarily be regarded as incapable of work through some specific disease or bodily disablement, and a certificate from the medical authorities of the sanatorium that he is receiving sanatorium benefit as an inmate is sufficient proof that the statutory conditions are fulfilled.

The reason we have for thinking that this case is important lies in the fact that the special provision for the payment of sickness benefit to voluntary hospitals in respect of persons without dependants, where an arrangement has been made with an approved society, arises under the same section of the Act as that which relates to persons without dependants who are inmates of sanatoria. There may be circumstances in which, in spite of any general agreement that may have been made, an approved society might resist a claim for the payment to the hospital of the sum equivalent to the sickness benefit. In such a case the decision above referred to would appear to be useful as indicating that the title to the payment is independent of the rules of the society, as they would apply to the members, and that a certificate from the medical authorities would be sufficient proof of incapacity for work.

